# Effects of Individuality, Education, and Image on Visual Attention: Analyzing Eye-tracking Data using Machine Learning

**DOI:** 10.16910/jemr.12.2.4

**Published:** 2019-07-16

**Authors:** Sangwon Lee, Yongha Hwang, Yan Jin, Sihyeong Ahn, Jaewan Park

**Affiliations:** Yonsei University, Seoul, South Korea; University of Michigan, Ann Arbor, United States; Qingdao University of Technology, Qingdao, China

**Keywords:** Eye tracking, visual attention, individual differences, art perception, architectural design, machine learning, classification, region of interest

## Abstract

Machine learning, particularly classification algorithms, constructs mathematical models from labeled data that can predict labels for new data. Using its capability to identify distinguishing patterns among multi-dimensional data, we investigated the impact of three factors on the observation of architectural scenes: individuality, education, and image stimuli. An analysis of the eye-tracking data revealed that (1) a velocity histogram was unique to individuals, (2) students of architecture and other disciplines could be distinguished via endogenous parameters, but (3) they were more distinct in terms of seeking structural versus symbolic elements. Because of the reverse nature of the classification algorithms that automatically learn from data, we could identify relevant parameters and distinguishing eye-tracking patterns that have not been reported in previous studies.

## Introduction

In the design research community, the benefit of using eye-tracking is distinct: it provides quantifiable information about a viewer’s visual attention in a non-intrusive manner. Its significance is based on the fact that the visual appearance of a product plays a critical role in consumer response (1) and on the hypothesis that eye-tracking data instantly externalize what people think (2) or aim to accomplish ( 3).

Eye-tracking is subject to certain restrictions though: processing eye-tracking data computationally has no universal standard (4), the link between eye-tracking measures and those of domain problems is hidden (5), experimental setups may not fully represent actual practice ( 6), and the quality of analysis often depends on the ability to build customized software (7). However, eye-trackers have been shown to provide objective measures that can be associated with high-level design problems, such as usability (8), training effects (9, 10), preference ( 11), and cultural influences (12).

Eye-tracking data have been linked to design problems through layers of mediating parameters. Human eye movement consists primarily of two phenomena: 1) fixation—a relatively stationary period of eye movement and 2) saccades—rapid movements between fixations. The low-level parameters, e.g., fixation duration, fixated positions, and saccade amplitude, have been combined as quantitative indicators of a specified design problem. For example, changes in fixated positions were related to the specified task (13), and the distribution of gaze durations (cumulative fixation duration within a cluster) has been found to quantify the difference between individuals with and without artistic training (9). The mean fixation duration and saccadic amplitude have been established to encode individualities (14), and the shapes of reading patterns have demonstrated the effects of cultural background ( 12).

The limitation of this practice is that the determination of the criterion parameters for a specified problem is not always straightforward or successful. That is, the selected eye-tracking parameters were often not effective indicators of the target effect. The consequence is the lack of agreement on the mapping between parameters and design problems (15), and studies whose findings partially support or lie outside the scope of the initial goal (11, 16, 17, 18, 19). One major cause of such phenomenon is the difficulty of dealing with multi-dimensional data; it is beyond human intuition to compare multiple high-dimensional data simultaneously. For example, we can visually inspect the scanpaths of two images, but comparing multiple scanpaths in two groups is significantly more challenging (20). We can compare mean fixation durations at once, but quantifying the differences of fixations over a period of time allows for multiple parameterizations, which makes it more complicated to gain insights and test hypotheses quickly. A more profound task is to relate the parameters embedded in high-dimensional eye-tracking data to higher-level domain problems. The total number of combinations of parameters grows exponentially with the dimension of the parameter, and finding the relevant eye-tracking parameters through iterative testing appears to be prohibitively inefficient. It requires a more effective method to measure the relative impact of eye-tracking parameters and detect hidden patterns.

According to Arthur Samuel, machine learning is a field of study that gives computers the ability to learn without being explicitly programmed ( 21). By virtue of its capability to identify trends and make predictions from multi-dimensional data, machine learning has significant potential for detecting new patterns and verifying existing propositions in eye-tracking studies. Greene, Liu, & Wolfe (22) and Borji & Itti (23) applied a classification algorithm to eye-tracking data labeled with task information and investigated the statistical foundation of the observation that the given task affects eye-tracking patterns (13). The key advantage of machine learning lies in the order of the process; it first learns from data and then identifies the parameters relevant to the classification, rather than first predicting the potential parameters and then verifying their impact. From this perspective, a classification algorithm is a reverse approach that can identify the relevant parameters more effectively than a forward-based one where the discovery of links between eye-tracking parameters and the target effect depends heavily on the initial choice of candidate parameters (23).

Motivated by the opportunities that machine learning offers, our study intends to evaluate the impact of three factors associated with viewing architectural scenes: individuality, education, and stimuli. Among the factors exogenous and endogenous to the participating individual, we designed the experiment such that eye-tracking data constituted the combined effect of natural tendency, architectural training, and image content. First, the presence of eye-tracking parameters unique to an individual has been studied extensively (14, 19, 22, 24, 25, 26). We explored new eye-tracking parameters that are likely to identify an individual from a larger dataset. Furthermore, the art and design community has been paying significant attention to distinguishing between “trained” and “untrained” eyes (9, 10, 16, 18, 19, 27). According to the notion that evaluative discrepancy in architecture is particularly expensive (28), we aimed to identify, quantify, and visualize patterns that distinguish between majors and non-majors of architecture-related disciplines. Finally, it has been reported that the presence of image content indicative of the specified task alters what people attend to (13, 22, 23, 29, 30). One of our primary focuses was the impact of image stimuli in relation to individuality or educational background. When classifications of an individual or major/non-major across all image stimuli failed to predict the identity, we compared the classification accuracy of each image and looked for the key image features that distinguished an individual or educational background.

## Background

In art and design research, eye-tracking data have been used as a quantifiable measure, an objective indicator, and scientific evidence of various aesthetic rules and design heuristics. In art research, one primary question was the manner in which trained artists behave differently from novices. According to Berlyne’s (31) notion of diverse vs. specific exploration, Nodine et al. (9) assumed that artists shift from specific to diverse exploration when the symmetry of aesthetic composition breaks. The hypothesis was verified by artists’ dispersed, shorter gaze durations at asymmetric compositions to which non-artists were less sensitive. In subsequent research, Vogt & Magnussen (27) found that artists pay more attention to structural aspects than to individual elements. Miall & Tchalenko (32) focused on the actual process of painting by combining an eye-tracker with a hand-tracker and identified three distinct patterns: initial prolonged attention to the model, rapid alternation of attention between the model and the canvas for sketching, and practice strokes on the canvas. They proposed fixation stability, fixation duration, and targeting efficiency as parameters for defining the artist’s eye skills and eye–hand coordination.

In design discipline, the eye-tracking research diversified by its sub-disciplines. In the product design domain, researchers have explored the use of the eye-tracker as a tool for understanding user preference within the entire product development cycle. Using the effectiveness of the eye-tracker for measuring user attention (33), Kukkonen (18) explored the connection between the attended area and product preference. Reid et al. (11) used eye-tracking data to corroborate survey information that investigated the influence of product representation on user selection, and Köhler, Falk, & Schmitt (34) proposed that eye-tracking aids the extraction of the visual impression and the emotional evaluation as part of the Kansei engineering process. In the visual communication domain, numerous studies have addressed the usability of 2D graphical user interfaces. Nielson & Pernice (8) used a large set of eye-tracking data to produce design guidelines for webpage design, and Dong & Lee (12) externalized how cultural background affects webpage reading behavior using eye-tracking scanpath maps. Prats, Garner, Jowers, McKay, & Pedreira (35) demonstrated that eye-tracking parameters can indicate the moment when shape interpretation occurs, and Ehmke & Wilson (15) listed the eye-tracking parameters relevant to various web usability problems. Two eye-tracking studies in the architecture domain have investigated the role of architectural elements and the impact of architectural training on viewing architectural scenes (16, 19). A study in the fashion design domain revealed how designers and non-designers view differently in the context of participatory design (10).

Occasionally, design research that used eye-tracking data exhibited variance in the level of success, i.e., differences in the number of objectively verified hypotheses and proposed hypotheses. The characteristics of these studies are the absence of analysis on the proposed questions, lack of quantitative reasoning, and high rate of unexpected findings. For example, Koivunen et al. (17) initially intended to reveal the influence of design education and rendering style; however, they observed behaviors during the first impression and different fixation durations per task. Kukkonen (18) measured gaze data, preference scores, the most favored product, and individual evaluations and concluded that there is negligible correlation among them. Reid et al. (11) identified that a long fixation duration can indicate either of high and low preferences, but did not provide statistical evidence or an in-depth analysis. Lee et al. (19) identified potential eye-tracking parameters for differentiating individuals that were not part of their original research questions. Occasionally, an unexpected factor, e.g., image size (18) and presentation order (11), was the source of the failure, but a more fundamental cause appears to be the inability to predict the affecting parameters. Whereas high-dimensional eye-tracking data enable a large set of parameter combinations, their connection to high-level design issues is not revealed until we test them.

Recently, two papers have reported controversial opinions on the observation of Yarbus (13) that the specified task affects the eye-tracking pattern. Greene et al. (22) displayed 64 images to 16 participants with four tasks but the correct prediction rate was only marginally higher than random chance (27.1%, 95% CI = 24–31%, chance = 25%). Using the same data, Borji & Itti (23) disputed the conclusion with a significantly higher prediction rate (34.12%). The element that differentiates their methods from previous ones was the adoption of machine learning, in particular a classification algorithm. More traditional approaches would have selected a set of indicative eye-tracking parameters and tested whether they fluctuate by a significant margin as the specified tasks differ. Rather, they constructed a prediction model using training data and analyzed its performance by comparing the predicted task with the actual task using validation data. The prediction model essentially draws boundaries between eye-tracking data with different tasks within the multi-dimensional parameter space. Its performance depends on how clearly the model can detect boundaries among training data and the extent to which the logic for dividing the training data is applicable to the validation data. The key difference between Greene et al.’s (22) and Borji & Itti’s (23) studies was the selection of the classification method for constructing the prediction model, i.e., a linear discriminant vs. the RUSBoost classifier.

In this study, we aimed to evaluate the impact of three factors, i.e., individuality, educational background, and image stimuli, by using machine learning to explore multi-dimensional data. Regarding individuality, the question has been whether endogenous eye-tracking parameters consistent across different viewing conditions exist. The motivation was to know (1) the extent to which endogenous factors affect eye-tracking patterns, and (2) the potential connection with neural substrates, such as ADHD, dementia, memory (14), visual search performance (25), and visual recognition strategy (26). Andrews & Coppola (24) found that the mean fixation duration and saccade amplitudes formed a linear relationship in active and passive viewing tasks. Castelhano & Henderson (14) demonstrated that these parameters are stable across differing image content, quality, and format. Greene et al. (22) succeeded in predicting the identities of eye-tracking data using machine learning with significantly higher probability than random chance (26% vs. 6.3%). Recently, Lee et al. (19) proposed the existence of additional patterns unique to certain individuals based on visual inspection. In this study, we searched for more fingerprinting patterns with higher predictability. Second, previous studies have found that groups of individuals with and without certain educational training differed in exploration patterns or cumulative fixation durations on the designated area of interest. The group with educational training focused more on the background or structural relationships among individual elements (9, 16, 27) and on image content that was relevant to the focus of their training (10, 19). Observing that only a few eye-tracking parameters have been associated with group characteristics (9, 10, 16, 19), we explored additional parameters distinguishing between major and non-major students of architecture discipline. Finally, the impact of image stimuli varied in different decoding tasks. Although it was not sufficiently strong to affect individual decoding (14), image content with diagnostic information relevant to the specified task was crucial in task decoding ( 23). Image contents such as symmetry (9), background complexity (10), and inclusion of architectural elements (19) were found to affect the decoding of educational background. Our focus in the case of image stimuli was to identify particular image content that attracts a particular individual or major/non-major.

## Methods

We used the data generated by Lee et al. (19), which is publicly available (http://bit.ly/2eqb4TV), as input data. To observe the effect of architectural training on visual attention, they recorded 10-s eye-tracking patterns of 71 major/non-major participants (39 majors and 32 non-majors) on 14 images with certain architectural elements (Appendix). The data consist of eye positions sampled with a frequency of 60 Hz in normalized coordinates: a screen space with a size of 1.0 (width) by 0.74 (height). A fixation was defined as a group of sample points whose diameter does not exceed 0.02 in normalized length and 300 ms in time between the first and last one. All other events, such as glissades and smooth pursuits as well as saccades in the traditional sense were collected into single ‘saccade’ category in our study, following the **I**dentification by **D**ispersion **T**hreshold algorithm (Figure 1) ( 36). Hence, the definition of saccade in this paper is broader than more typical and conventional definition of saccade usually between 30-500 deg/s.

**Figure 1.**
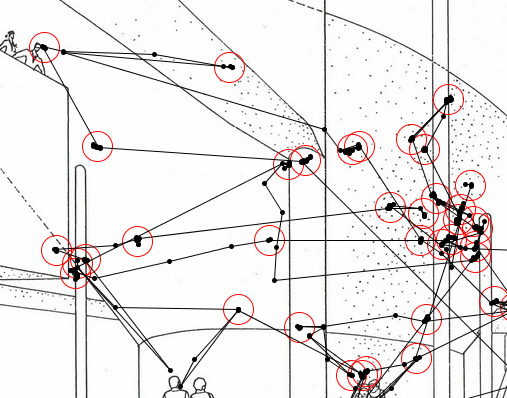
Fixations from eye positions using the I-DT algorithm

Rather than establishing candidate parameters and verifying their statistical significance, we applied machine learning to identify the distinguishing parameters and their patterns that characterize individuals and majors/non-majors. Regarding the data features for decoding individuals and majors/non-majors, we considered characteristic patterns such as oscillating movements and the extent of fixation over time proposed by Lee et al. (19), as well as well-established endogenous parameters such as total fixations, mean fixation duration, and mean saccade amplitude (22). To understand the impact of image stimuli, we also included a fixation map representing the cumulative fixation durations on each of the 10 × 10 cell grids within the image area. The complete list of features is as follows:

(1) Fixation data comprising average fixation duration, total fixation count, average saccade length, and total saccade count

(2) Fixation histogram data whose bins represent different ranges of fixation duration

(3) Saccade histogram data whose bins indicate ranges of saccade lengths

(4) Velocity histogram data whose bins are ranges of normalized lengths between adjacent points sampled at 60 Hz

(5) Fixation map data representing cumulative fixation durations on a 10 × 10 grid

Velocity histograms store normalized lengths traveled in 1/60 of a second. Because a 24.98 cm × 18.61 cm screen was placed 50 cm from the participant, velocity *v* (s^-1^) can be converted to a degree in the visual angle by using 2 tan^-1^ ( *v*/2 × 24.98/50) (deg/s). The histogram consisted of 14 bins, 1 special bin reserved for zero velocity, and 13 bins for the rest. The ranges of 13 bins were determined by first sorting the data and then dividing them into 13 groups of equal sizes. Fixation and the saccade histogram consisted of 10 bins of equal sizes with no special bin.

In our study, we performed the classification by using each individual or major/non-major labels. Because the performance of the prediction model can vary widely according to the selected classifier ( 22, 23), we compared the results of three classifiers: decision tree, a support vector machine (SVM) with a linear kernel (SVM.LinearSVC), and an SVM with a radial basis function kernel (SVM.SVC) implemented with the Python machine learning package ( www.scikit-learn.org). While SVM with a linear kernel was the choice of previous eye-tracking research with high-dimensional data (22), we tested a radial kernel because it may perform better for lower-dimensional data with feature selection. Decision tree was useful to compare the importance of different features. For SVM classifiers, we applied feature selection by using the extremely randomized tree (37) with [1.7, 1.8, 1.9, 2.0, 2.1] × mean as the threshold, [10, 30, 50, 70, 90] as the max depth, and [2, 5, 10, 15, 20] as the min_samples_split. For hyper-parameter tuning, [0.25, 0.5, 1, 2, 4] was used as the C value. For decision tree, we used [gini, entropy] as the criterion, [10, 20, … 80] as the max_depth, [auto, sqrt] as the max_features, [1, 2, …, 10] as the min_samples_leaf, and [2,3, .., 10] as the min_samples_split.

To decode the individual identities of the eye-tracking data, we compared the correct prediction rate (accuracy) against the random chance level (1 / 71 individuals = 1.41%). Different classifiers were compared to determine the most optimum result. To split the entire data into training and validation datasets, we adopted the leave-N-out selection scheme; 71 samples from the individual-image pairs formed the validation set and the remaining (14 - 1) × 71 samples formed the training set. With each of the 14 iterations, we chose one image out of 14 images to form a validation set. This folding scheme allowed no sample in the testing set see the target image of the validation set, ensuring that the prediction of validation set is based solely on endogenous factor (individuality) by excluding the effect of exogenous factor (image). An exhaustive alternative would have been to iterate over all the 14^71^ training/validation set combinations. To decode majors/non-majors, we divided the eye-tracking data of all the participants (14 images × 71 participants) into 70/30, i.e., 70% for the training dataset and 30% for the validation dataset. As with individual decoding, we compared the performance of different classifiers averaged over 14 iterations. To estimate the statistical significance, we adopted one-way ANOVA using 14 data samples against the chance level (50%) in accordance with Greene et al. (22). Please note that the histogram bin ranges were recalculated for each iteration by using the data samples in the training set only. This was done to make sure that the validation set had no effect on feature extraction (Table 1).

Finally, in order to investigate the effects of image stimuli, we classified 71 participants’ data for each image into major/non-major groups and identified the image content that contributed to high correct prediction rates. We ran 71 iterations per image; in each case, one of the 71 data samples formed the validation dataset and the remaining formed the training dataset. Decoding an individual per image was not feasible because there was only one sample from each participant per image, preventing division into training/validation datasets.

## Results

**Table 1:** The range of each bin averaged over 14 iterations.

Bin	1	2	3	4	5		
Fixation duration histogram	0.050 ± 0	0.111 ± 2.01e-3	0.169 ± 5.34e-4	0.201 ± 6.76e-4	0.222 ± 1.45e-4		
	**6**	**7**	**8**	**9**	**10**		
	0.233 ± 2.59e-5	0.250 ± 2.77e-17	0.283 ± 1.62e-6	0.476 ± ±4.11e-3	∞		
**Bin**	**1**	**2**	**3**	**4**	**5**		
Saccade length histogram	0.020 ± 4.81e-5	0.027 ± 8.72e-5	0.035 ± 1.37e-4	0.045 ± 2.21e-4	0.057 ± 2.81e-4		
	**6**	**7**	**8**	**9**	**10**		
	0.072 ± 3.97e-4	0.094 ± 4.82e-4	0.125 ± 7.6e-4	0.184 ± ±1.36e-4	∞		
**Bin**	**1**	**2**	**3**	**4**	**5**	**6**	**7**
Velocity histogram	0.0 ± 0.0	7.79e-4 ± 4.37e-6	1.27e-3 ± 5.65e-6	1.79e-3 ± 7.86e-6	2.38e-3 ± 1.36e-5	3.08e-3 ± 1.63e-5	4.03e-3 ± 2.22e-5
	**8**	**9**	**10**	**11**	**12**	**13**	**14**
	5.39e-3 ± 3.46e-5	7.62e-3 ± 5.64e-5	0.012 ± 9.67e-5	0.0224 ± 2.46e-4	0.0456 ± 4.68e-4	0.0947 ± 7.49e-4	∞

### Decoding Individuals

Before matching various classifiers with different data features, we applied a decision tree classifier to obtain the relative importance of data features. The overall results indicated that the fixation data and velocity histogram were more effective for individual decoding than the others (Figure 2), with a higher correction prediction rate (967/7100 = 13.62%) than the chance level (1/71 = 1.41%). Figure 2 visualizes that the velocity histogram data (yellow), particularly the earlier bins, exerted the highest importance, followed by the fixation data (light blue). The importance of the fixation histogram (orange) and saccade histogram (gray) were lower than these two features. The fixation map data (dark blue) displays 10 peaks, whose maxima are higher near the middle (third to seventh peaks) than both ends. Each peak represents the longest fixation duration of each row in a 10 × 10 cell grid. The higher peaks near the middle indicate that cells around the center of an image were a better indicator of the identity of the individual.

**Figure 2.**
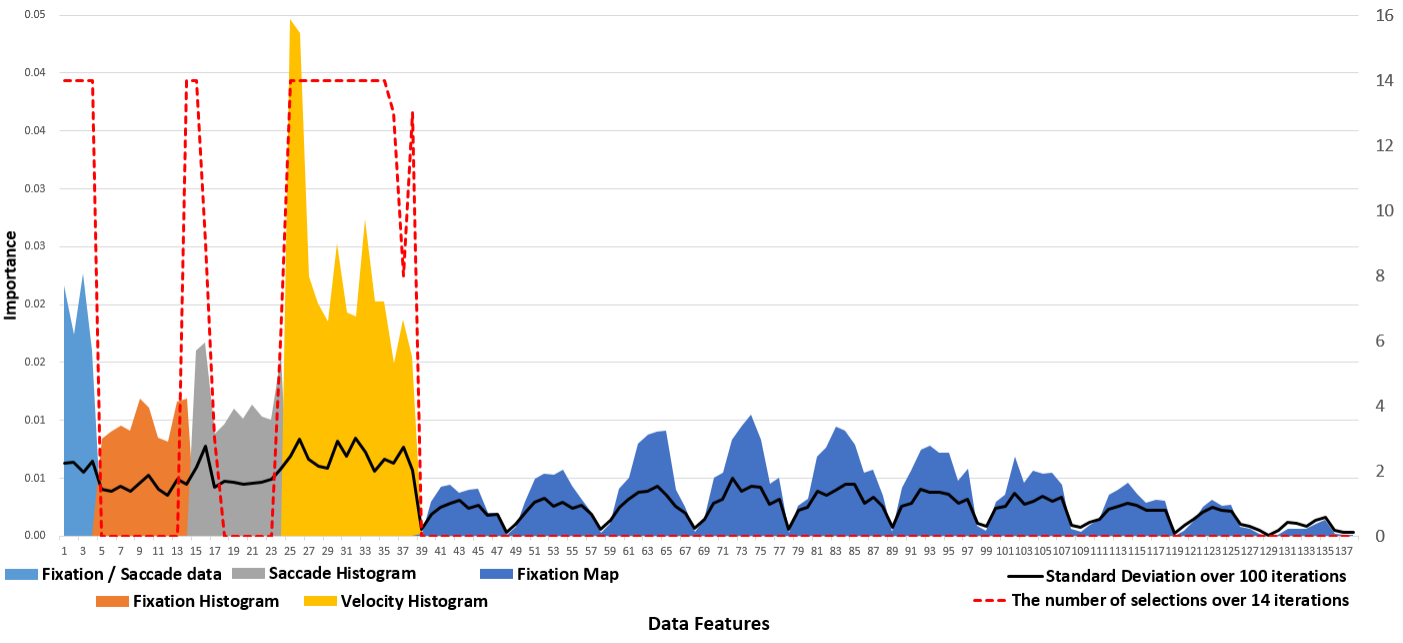
Importance of data features during individual classification. The indices on the x-axis correspond to the fixation data (1-4), fixation histogram (5-14), saccade histogram (15-24), velocity histogram (25-38), and fixation map data (38-138). Red dots indicate the number of selections over 14 iterations by feature selection.

**Table 2:** Correct prediction rate (accuracy) of different classifiers averaged over 14 iterations.

Classifiers	Accracy
Decision Tree	12.47 ± 4.19
SVM.LinearSVC	25.25 ± 3.82
SVM.SVC	31.09 ± 8.22
	65.29 ± 7.21 (top 5)

Table 2 presents the results of different classifiers obtained by using feature selection and hyper-parameter tuning on all data features. Whereas the decision tree exhibited an average correctness of approximately 14%, the LinearSVC classifier performed better, and the SVC classifier generated the highest correct prediction rate. The features chosen by feature selection over 14 iterations matched well with those having high importance in the preliminary testing run using the histogram ranges averaged over all folds (Figure 2, red dotted line). The top-five correct prediction rate with the SVC classifier was 65.29%, implying that the prediction rate doubles if we permit up to five guesses per individual.

### Difference between Individuals

Using the classification results with the best options (SVM.SVC classifier), we compared the correct prediction rate of each individual. Figure 3 (left) shows that the majority of the participants had a correct prediction rate higher than random chance (1.41%). The average correct prediction rate of the non-majors (33.3%) was higher than that of the majors (29.3%). Figure 3 (right) shows a confusion matrix that visualizes the correct and incorrect predictions. The x- and y-coordinates or each dot represent true and predicted individual for a specified data, and the bright diagonal line indicates the overall success of individual decoding. A few dots off the diagonal line are incorrect predictions, and the higher density of warm dots in the lower right quadrant is indicative of the high prediction rates of non-majors than majors.

**Figure 3.**
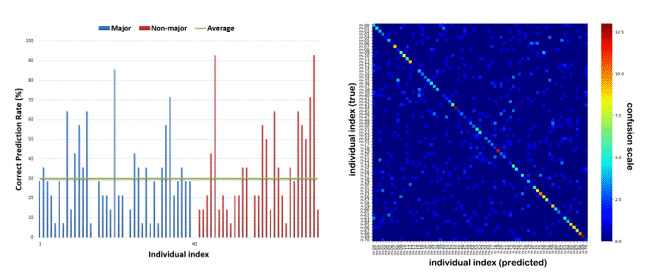
Correct prediction rate per individual (left) and corresponding confusion matrix (right).

### Consistency across Images

Figure 4 is a graph showing the velocity histogram per individual for all the images. It represents eight participants’ data with the highest and lowest correct prediction rates. The highest rates were 92.86% (Figure 4(a,b)), 85.71% (Figure 4(c)), and 71.43% (Figure 4(d)); the lowest were 7.14% (Figure 4(f)) and 0% (Figure 4(e,g,h)). Each Figure contains 14 colored lines, representing data from all the 14 images. The most prominent difference between the two groups was the consistency across images; the first four participants, particularly Figure 4(b), tend to have more narrowly clustered lines than the others. Moreover, as the early bins exert larger impact for individual decoding (Figure 2), the level of convergence at bin 1 appears to have contributed to the higher prediction rate of Figure 4(d) than that of Figure 4(f,h) notwithstanding their overall similarity. The highest performance in Figure 4(a) seems to be explained by its uniqueness of pattern among the participants; this is further supported by the fixation data (Figure 5(d)).

**Figure 4.**
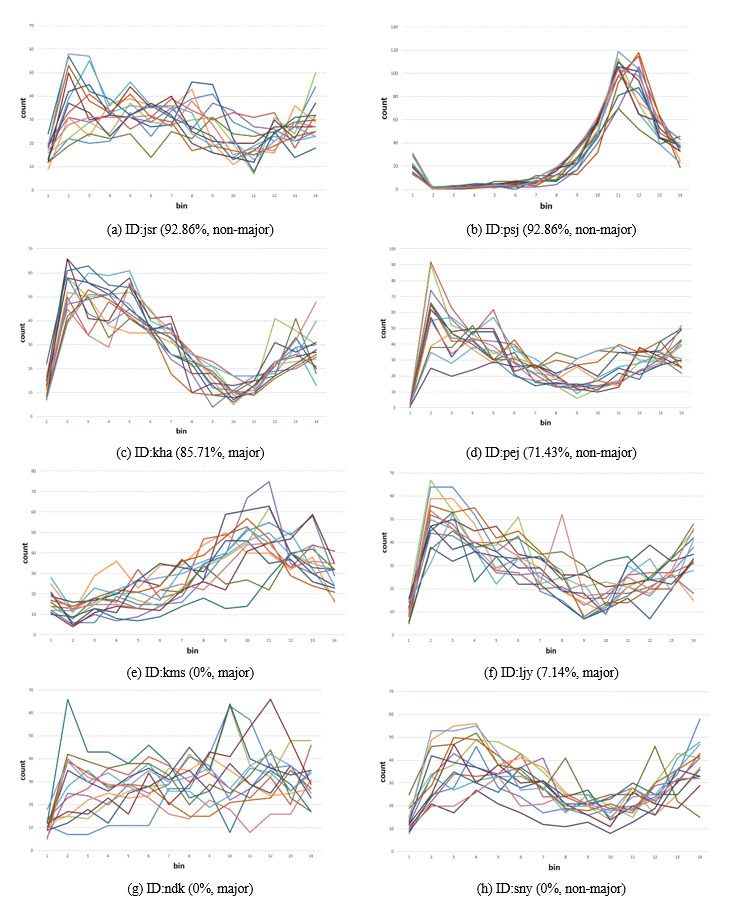
Velocity histogram of the individuals with top four prediction rate ((a)–(d)) and bottom four prediction rate ((e)–(h)), using the average bin ranges shown in Table 1.

**Figure 5.**
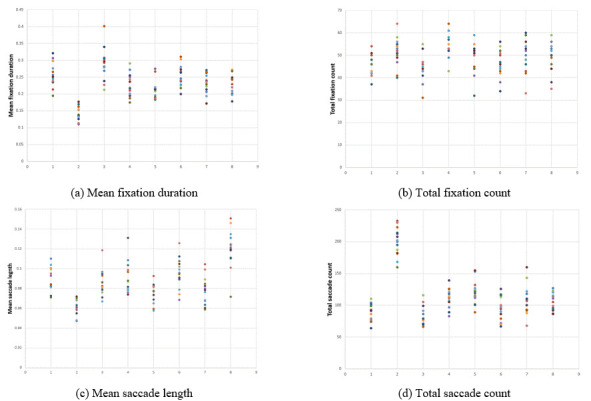
Fixation and saccade data features of the eight participants in Figure 4. 1–8 on the x-axis corresponds to (a)–(h) in Figure 4. The dots of the different colors represent the samples from the 14 images.

We could observe such consistency from the scanpath visualization as well. Figure 6 shows the actual eye-tracking patterns of the two participants (Figure 5(b,d)), whose lines with identical colors represent the same bin in the velocity histogram. We can infer from this that for each individual, the ratio between the numbers of the lines with the same color is more or less stable across the images. Moreover, as a unique sequence of colored lines repeats, it seems that it is not just the distribution of the lengths of these lines but also the order of their occurrence of them that carries individual character. For example, whereas a long red line and a set of shorter blue lines alternate in Figure 6 (left), there are numerous green dots in conjunction with the adjacent blue lines between the longer red lines in Figure 6 (right). Such an observation implies that we can have a better measure representing an individual character by incorporating both temporal (e.g., the order of lines) and spatial (e.g., orientation) properties.

**Figure 6.**
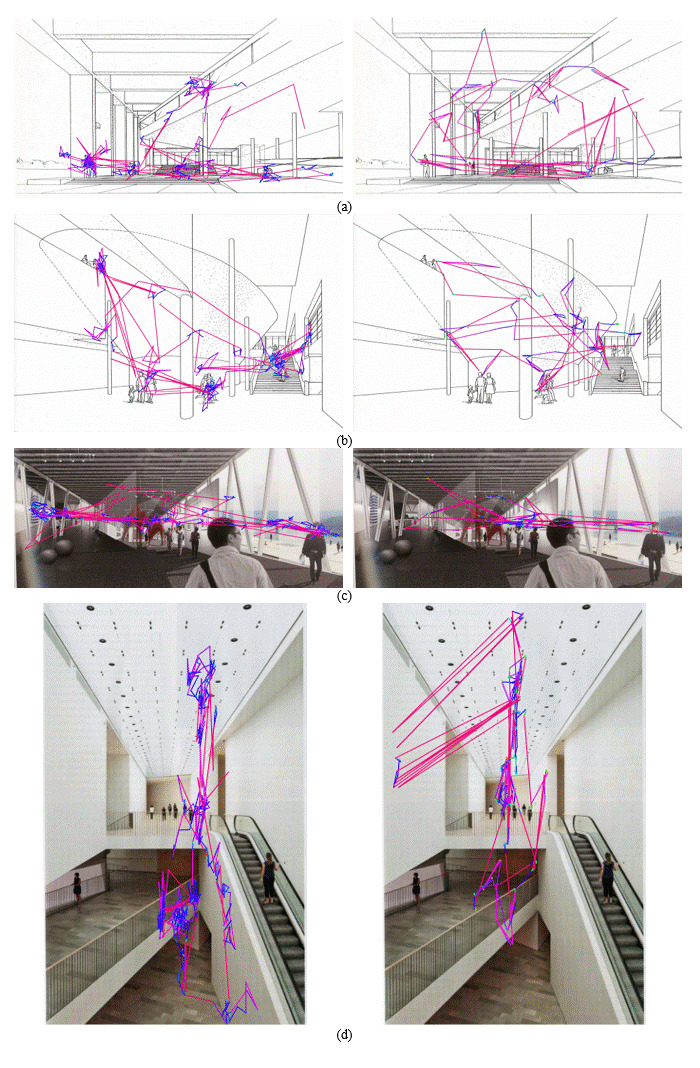
Scanpath visualization of individuals in Figure 3(b) (left column) and Figure 3(d) (right column).

### Decoding Majors/Non-majors

A preliminary run of the decision tree classifier for major/non-major decoding revealed that the distribution of importance over all the data features is similar to that of individual decoding (Figure 7). However, the standard deviation over 100 iterations exceeds the average values, indicating the unavailability of clear feature(s) containing the unique properties of the majors/n­­­on-majors.

**Figure 7.**
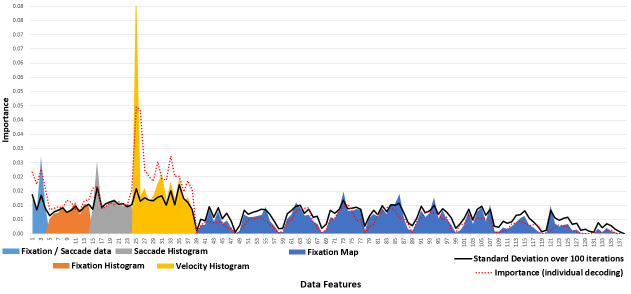
Importance of data features during major/non-major classification.

Next, we matched three classifiers with different data features. The best result (64.5 ± 7.16%) was from the SVM.SVC run with feature selection and hyper-parameter tuning on all data features, as in individual decoding. Whereas t-test verified a statistically meaningful difference (*p* = 2.73e-38) against the chance level (50%), non-majors exhibited significantly lower performance (57.32%) than majors (70.39%). When we plotted a 2D graph using the two features with the highest importance (Figure 8), the majors were more narrowly clustered than the non-majors with substantial overlap. We can infer that the shared area had been labeled as majors’ territory, and only the non-majors outside this territory were correctly predicted as non-majors.

**Figure 8.**
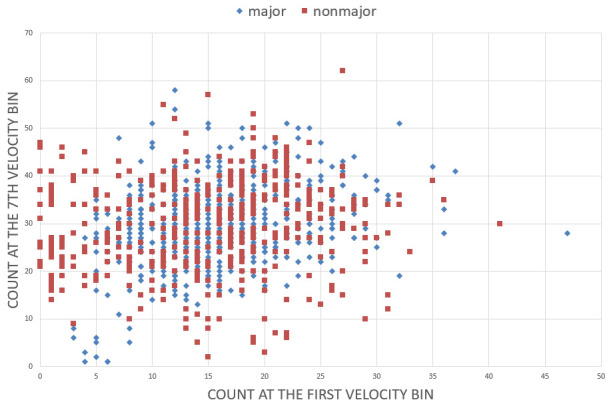
Distribution of the majors/non-majors by data points at the first bin (x-axis) and 7th bin (y-axis) of the velocity histogram.

### Influence of Image Stimuli

The major/non-major classification result was statistically meaningful, but not impressive, revealing that endogenous features were not good indicators. We then considered the final factor, i.e., the impact of image stimuli, with a focus on exogenous features as recommended in previous studies (9, 19). However, when we applied a fixation map data with a 10 × 10 grid, the overall prediction rate was significantly lower owing to the insufficient resolution. We could obtain comparable results by enhancing the 10 × 10 grid to a 20 × 20 grid (Table 3), using LinearSVC as a classifier because of its suitability with high-dimension data.

Table 3 illustrates that certain images are more effective in distinguishing between majors and non-majors than others. To identify the content receiving different level of attention, we focused on those image with a performance of 70% or higher and visualized the cumulative fixation time with red (major dominant) or green (non-major dominant) with transparency (Figure 9). The larger the difference, the brighter and more transparent the color became. We also marked the fixation time on each cell to differentiate between cells with equally high and low attention.

**Table 3:** Per image classification result with LinearSVC classifier on 20 × 20 fixation map data.

Img	1	2	3	4	5	6	7	Avg
Rate	61.8	70.6	61.8	52.9	50	64.7	58.8	**60.7 ± 7.95 (%)**
Img	8	9	10	11	12	13	14	
Rate	70.1	47.1	50	58.8	64.7	73.6	64.7	

**Figure 9.**
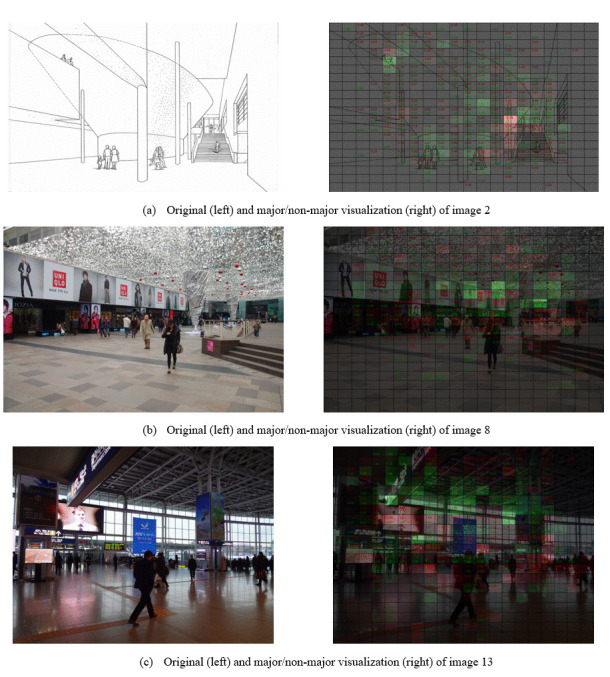
Original (left column) and major/non-major visualization (right column) of images 2, 8, and 13.

In Figure 10(a), both the majors and non-majors focused on human figures, but the non-majors exhibited marginally higher concentration (green box). We could observe larger differences at architectural elements with complex forms: conjunction between a column and a beam, the space between the stairs and a column, and the setback of the ceiling slab around a column. It appears that majors spend more time processing and interpreting structural ambiguities.

**Figure 10.**
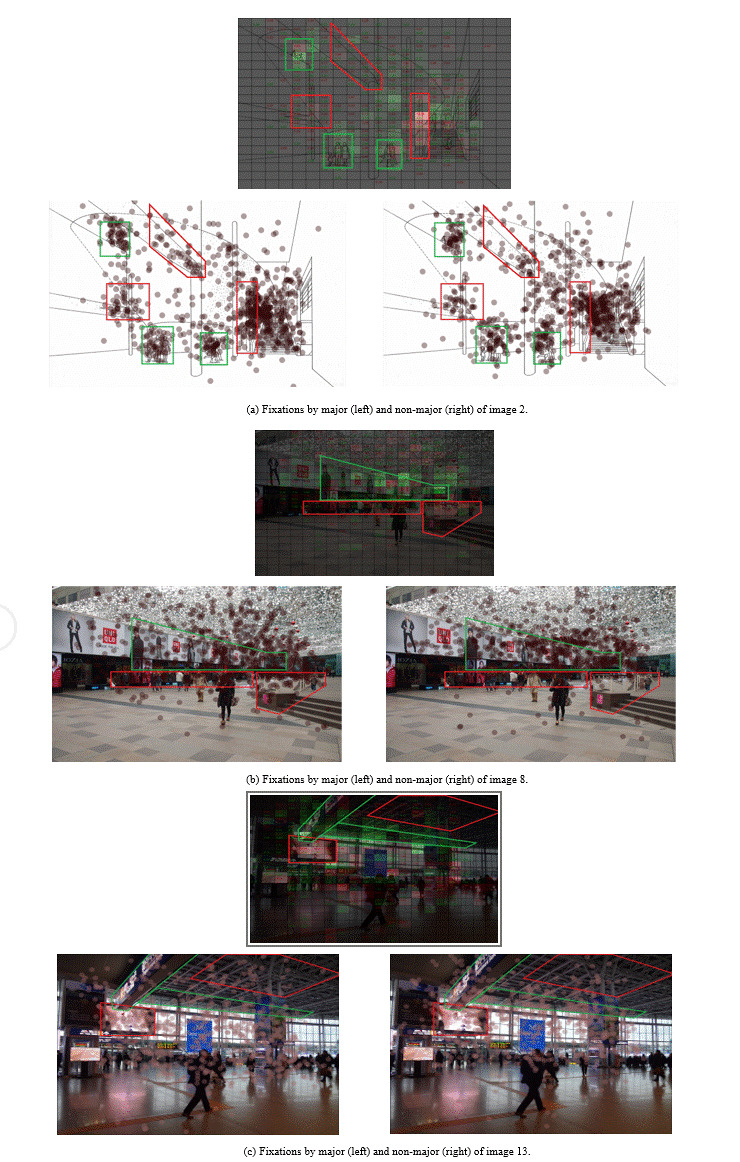
Visualization of areas receiving uneven attention by majors and non-majors.

In Figure 10(b), it is noticeable that the non-majors focused more on the commercial signboards, particularly those in the brighter upper area. The strength of focus tended to get intensified toward the vanishing point. On the other hand, the majors spread more to the darker region right below the commercial signboards and paid more attention to the complex shape of the stair rails.

In Figure 10(c), both the majors and non-majors paid attention to the LED display. However, the majors stayed longer near the larger screen, whereas more non-majors tended to read the direction sign. A subtle albeit visible difference was the uneven attention toward the roof truss structure. Whereas the non-majors concentrated most on the brighter side, where the window wall and the ceiling meet, some majors focused on the ill-lighted deeper area.

Overall, the majors exhibited a tendency to focus more on the structural elements, whereas the non-majors were attracted to signboards or human figures. The lighting condition and complexity of the shape appeared to play a role in the division of attention in that the darker and more complex a target element was, the longer the majors stayed than non-majors. However, an exception was that the majors paid stronger attention to the large LED screen area.

## Discussion and Conclusions

To investigate the impact of different endogenous and exogenous parameters on how we view architectural scenes, we applied a classification algorithm to multi-dimensional eye-tracking data obtained from students of architecture and other disciplines. We verified the effect of three factors, namely individuality, major/non-major, and image stimuli, on visual attention. The individual identity of the eye-tracking data was encoded in the velocity histogram, representing the distribution of the speed of eye movement measured at a fixed frame rate (60 Hz). The separation between the major and non-major groups was enabled using endogenous parameters, although it could be better explained by the differing sensitivities toward structural and symbolic image features.

Regarding individual decoding, the classification was successful using velocity histogram when the impact of other factors such as fixation duration and count was not as much significant. This is inconsistent with previous findings where the fixation duration and saccade length were consistent across different images (24) and the mean fixation duration, count, saccade amplitude, and coverage percent could classify individuals (22). An explanation is that our classification algorithm required more explicit distinction between individuals in a larger pool than previous forward- or reverse-based approaches (16 by Greene et al. (22) vs. 71 in our experiment). Therefore, we recommend using the velocity histogram for better individual decoding, in addition to the mean values of fixations and saccades. The use of the eye movement distances is not completely novel in eye-tracking research; Castelhano & Henderson (14) presented a profile of saccade distribution by length. However, their purpose was to demonstrate how natural saccade distribution could change according to the image types rather than its effectiveness in individual decoding.

The visual analysis of the velocity histogram revealed that a sequence of spatiotemporal pattern, rather than only the distribution of speed, was unique to each individual. Whereas a histogram could capture an aspect of such a pattern, it is not straightforward to determine which parameter can summarize such a feature more effectively. It appears challenging to (1) define the length of a sequence, (2) determine the tolerance of the variation, and (3) completely accommodate spatial disposition in a smaller parameter space. We consider that this venue of exploration has a potential for future research.

It is not evident why certain individuals exhibit stronger characteristics than others; in particular, the group of non-majors included a higher number of similar individuals than that of majors (Figure 3). The question is whether endogenous eye-tracking parameters are innate or acquired. Previous research has concluded that fixation and saccadic measures are natural properties determined by physical, neural, developmental, and psychological constraints (14) and that they are consistent across substantially different image contents (24) and tasks (25) and an 18-month period (26). Whereas our findings imply that a longer period of training involving visual construction affects endogenous parameters, we should also consider the likelihood that individuals with certain characteristics tend to select similar disciplines. An investigation of eye-tracking patterns over a period of educational training will help find the answer to this question.

Regarding decoding majors/non-majors, the classification was statistically significant using similar data features for individual decoding. However, the prediction rate was substantially higher for majors than for non-majors, as revealed by a 2D map whose x- and y-axes are two data features with the highest importance. The areas occupied by the two groups exhibited a large overlap, but because the majors were more narrowly clustered, the shared area had been marked as majors’ territory. It resulted in the incorrect prediction of non-majors in that area as majors. The map itself is a discovery of data features characterizing the randomness of a more heterogeneous group, but its level of distinction does not appear significant.

The analysis of image stimuli revealed that the level of attention of the majors and non-majors to certain elements differed. Whereas both groups tended to fixate on visually dense areas, the majors focused more on architectural elements (stairs, columns and beams, and truss structure) and the non-majors focused more on non-structural elements (commercial boards and entourage objects). It is noteworthy that the division was prominent where a feature exhibited a complex shape or was in low lighting condition. To summarize, whereas the majors aimed to resolve structural uncertainty, the non-majors were affected more by direct symbolic cue (9, 10). Its design implication is that architectural design should not only focus on organizing spaces but also consider the effect of symbols relative to visual attention. Considering that the training process is irreversible, user participation and an active use quantification method appears essential.

A limitation of the process here is that the classification performance depends highly on the resolution of the grid. In theory, a higher granularity always yields better classification results because it essentially creates more room for boundaries between different groups. Meanwhile, we also noticed a significant number of image features lying on the boundary. We recommend that a classification analysis based on boundary construction be interpreted and supplemented by other visual inspection methods. Another important point is that the reproducibility of our results will depend on the accuracy or precision of the measurement. We used raw data whose positions in normalized coordinates had a resolution in the order of 10e-4, and a timing in the order of 2.5e-6 ms, well below the frequency of the recording (60 Hz, 16.6667 ms). In our study, the first bin of the velocity histogram represents the “zero” distance between adjacent eye positions, and the existence itself along with its low to high variability across and within an individual is one proof of the soundness of the small scale data (Figure 4 and 5).

In conclusion, the application of machine learning to eye-tracking data revealed more data features unique to an individual and provided objective measures indicating the uneven attention between groups with and without educational training. Unlike previous forward-based approaches that test the effectiveness of the selected parameters, machine learning could automatically identify the distinguishing patterns from the candidate features in high dimensional spaces. However, it is also true that machine learning is not a panacea that can reveal all the hidden eye-tracking parameters. Not only did previous studies show the effectiveness of various parameterizations, but also histogram features in our study depended largely on researchers’ insights rather than blind application of machine learning. The problem proposed as future research – investigation of better methods for capturing the spatiotemporal nature and spatial distribution of eye movement – will also require trial and error of multiple hypotheses. In conclusion, the practice of forward-based searches of eye-tracking parameters will continue to exist in the near future, but the machine learning community will keep offering strong alternatives for exploring eye-tracking parameters more effectively and these alternatives would be worthwhile to consider.

## Ethics and Conflict of Interest

The author(s) declare(s) that the contents of the article are in agreement with the ethics described in http://biblio.unibe.ch/portale/elibrary/BOP/jemr/ethics.html and that there is no conflict of interest regarding the publication of this paper.

## Acknowledgements

This research was supported in part by Basic Science Research Program through the National Research Foundation of Korea (NRF) funded by the Ministry of Education (2014S1A5A8012349).

## Figures and Tables

**Figure 1:**
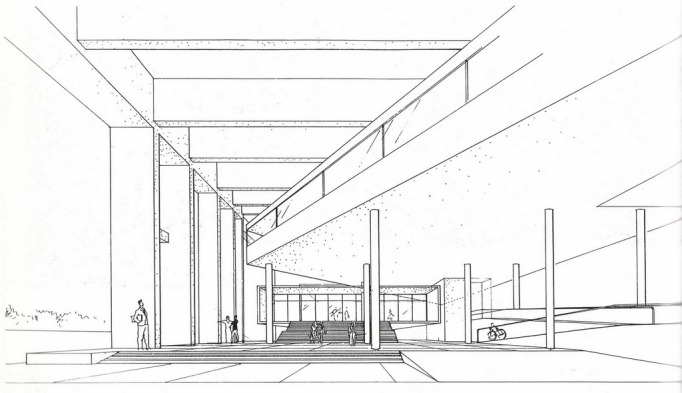
Kim H, Kim Y (1993) Musee d’architecture. Seoul: Baleon

**Figure 2:**
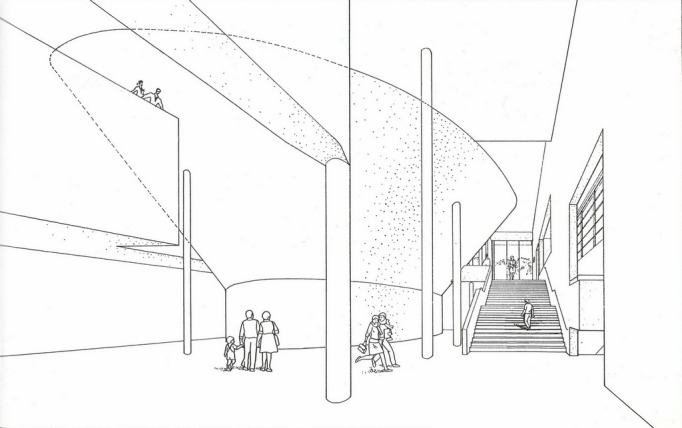
Kim H, Kim Y (1993) Musee d’architecture. Seoul: Baleon

**Figure 3:**
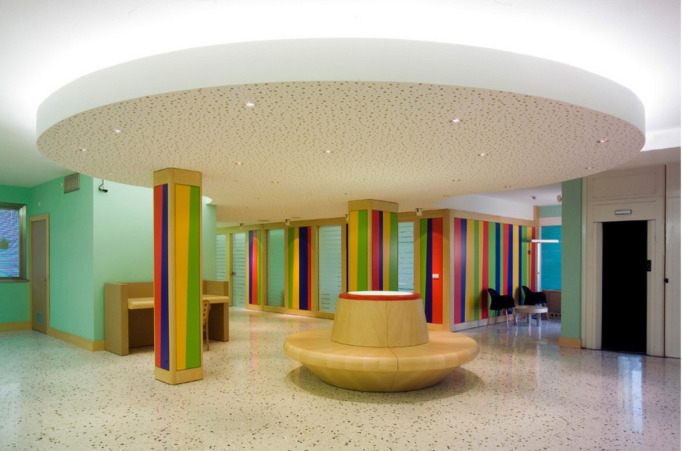
Archdaily, May 3, 2009. “Bank in Donoratico / Massimo Mariani”

**Figure 4:**
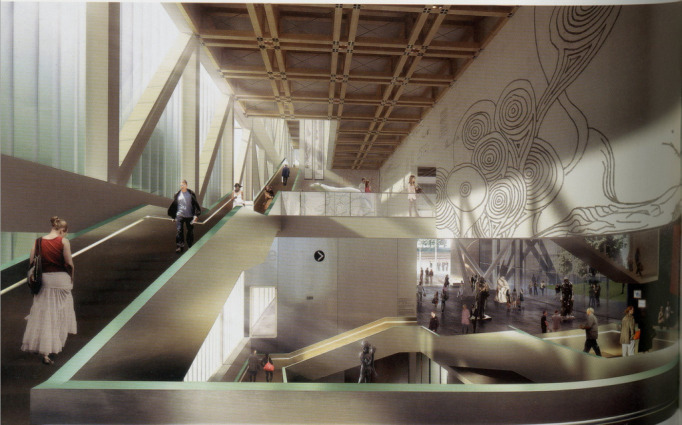
Musee National des Beaux-Arts du Quebec, OMA. Rendering by Luxigon

**Figure 5:**
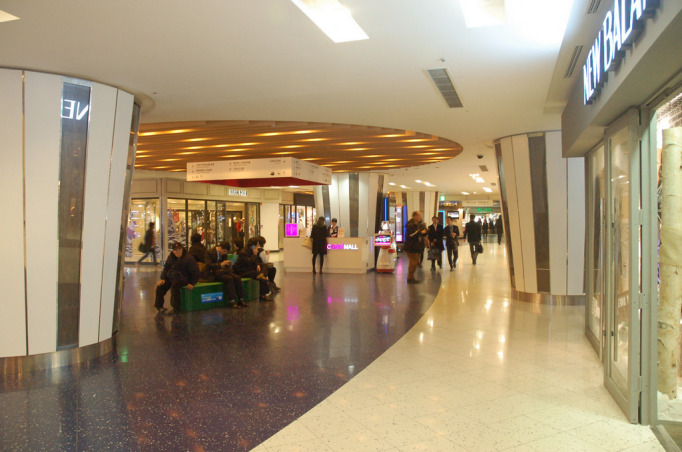
-

**Figure 6:**
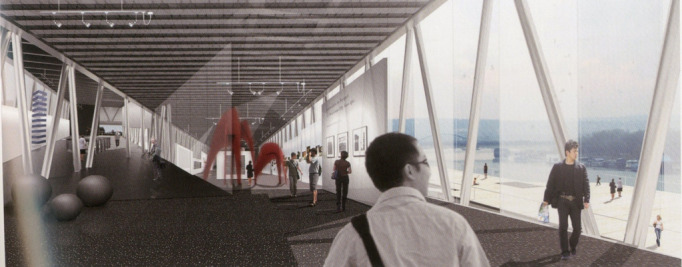
Archdaily, June 2, 2011. “Beton Hala Waterfront / dRN Architects” written by Hank Jarz. Architect: Max Nuñez

**Figure 7:**
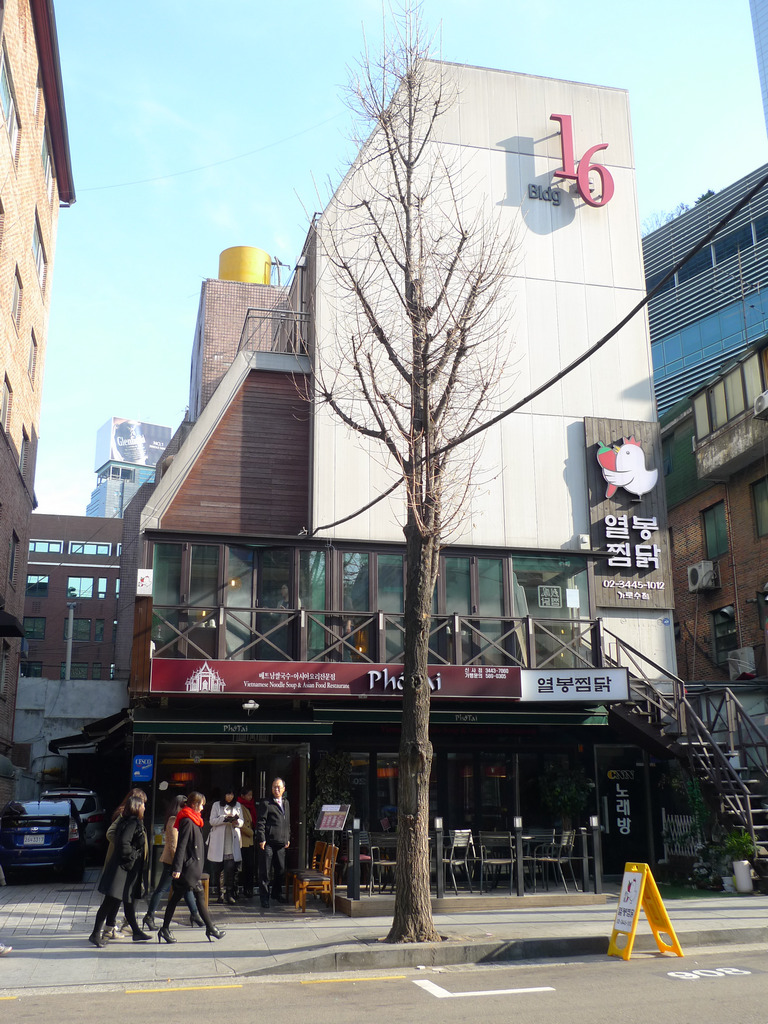
-

**Figure 8:**
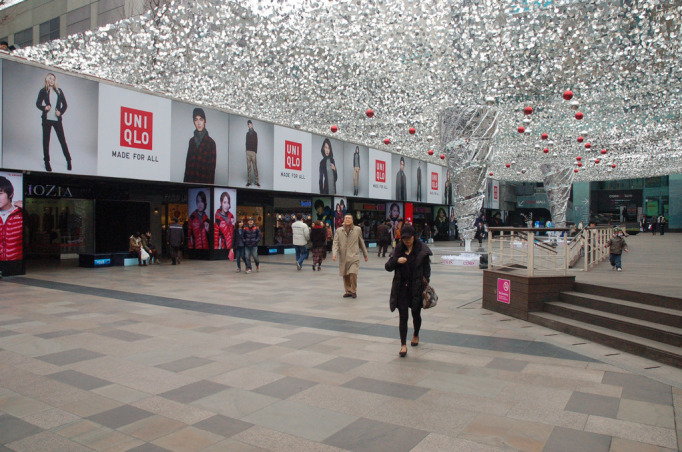
-

**Figure 9:**
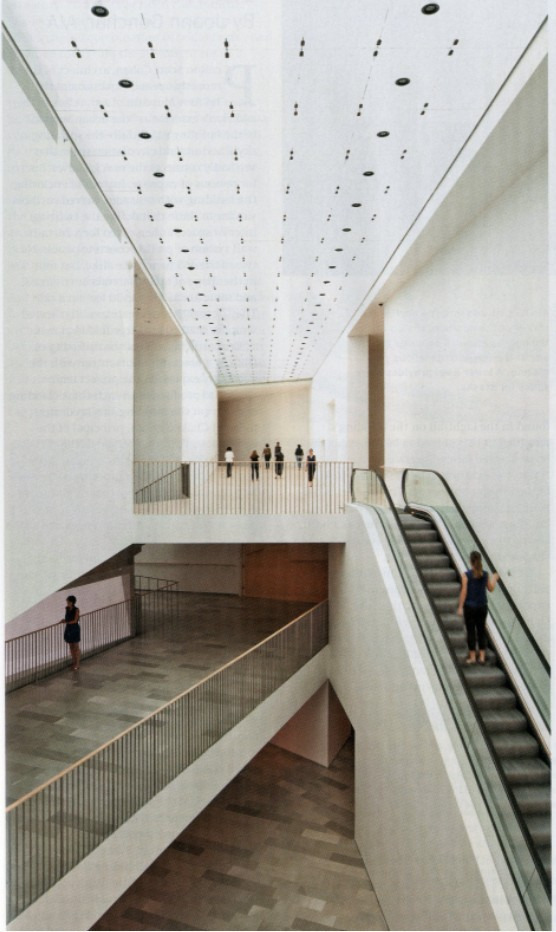
Architectural Record, November 2011, Tel Aviv Museum of Art p79, architect: Preston Scott Cohen

**Figure 10:**
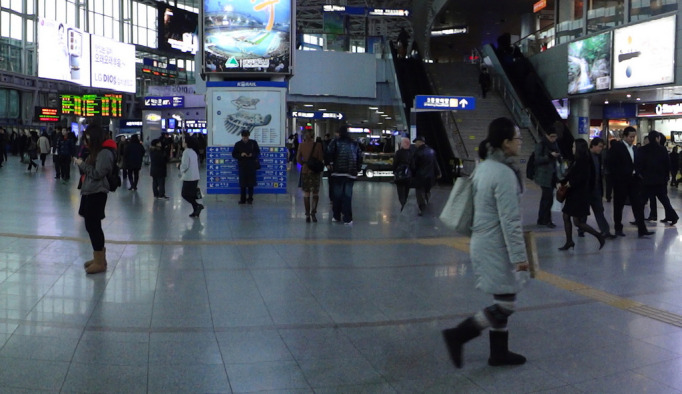
-

**Figure 11:**
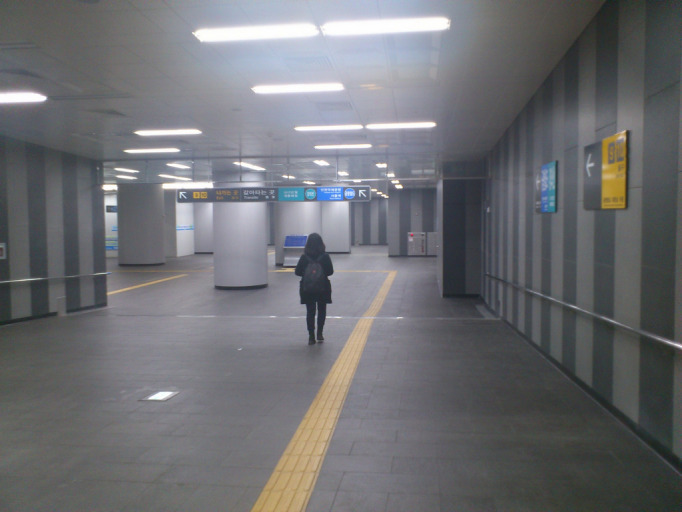
-

**Figure 12:**
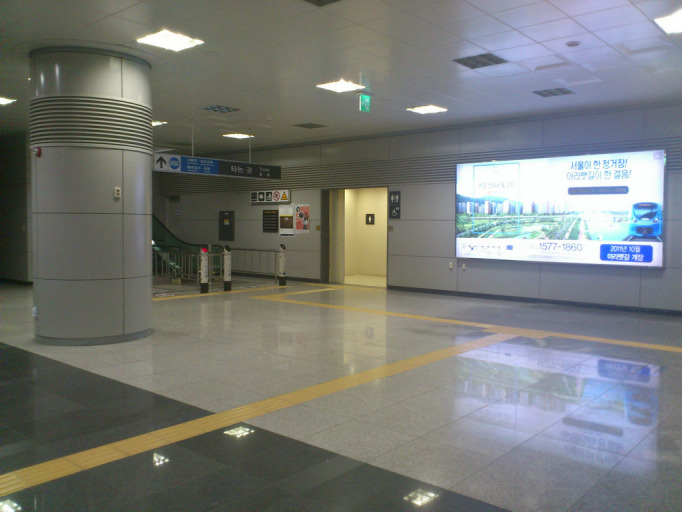
-

**Figure 13:**
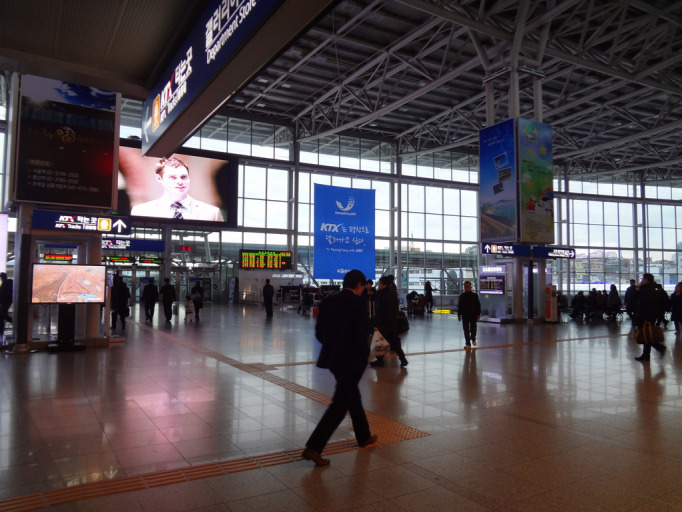
-

**Figure 14:**
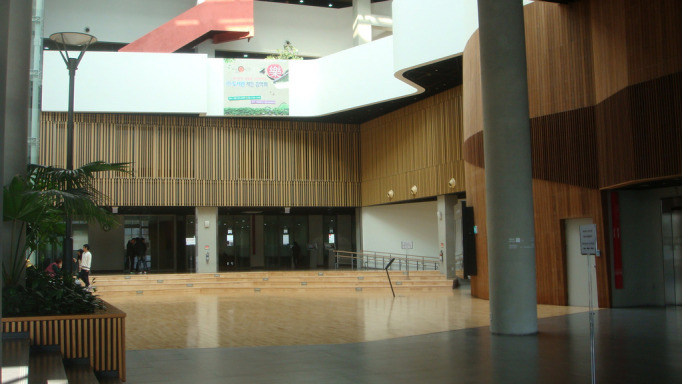
-
